# Duodenal subepithelial neuroendocrine tumor removed by endoscopic submucosal dissection using internal traction with magnets

**DOI:** 10.1016/j.vgie.2022.03.007

**Published:** 2022-04-23

**Authors:** Francisco Baldaque-Silva, Naining Wang, Masami Omae

**Affiliations:** 1 Division of Medicine, Department of Upper Gastrointestinal Diseases, Karolinska University Hospital and Karolinska Institute and Karolinska Institute, Stockholm, Sweden; 2 Department of Clinical Pathology, Karolinska University Hospital and Karolinska Institute, Stockholm, Sweden; 3 Division of Medicine, Department of Upper Gastrointestinal Diseases, Karolinska University Hospital and Karolinska Institute and Karolinska Institute, Stockholm, Sweden

**Keywords:** ESD, endoscopic submucosal dissection

## Abstract

Video 1 Endoscopic submucosal dissection of a duodenal subepithelial neuroendocrine tumor using internal traction with magnets.

Endoscopic submucosal dissection of a duodenal subepithelial neuroendocrine tumor using internal traction with magnets.

We report the case of a 57-year-old woman with multiple endocrine neoplasia type 1 who was referred to us because of the presence of a 15-mm subepithelial lesion in the descendent duodenum. Earlier endoscopic ultrasound and biopsies confirmed the diagnosis of neuroendocrine tumor in the submucosa. The patient was asymptomatic, blood test results were unremarkable, and there were no metastases on positron emission tomography/CT.

Endoscopic submucosal dissection (ESD) was chosen after multidisciplinary conference and with the patient’s informed consent.

ESD was performed with the patient under general anesthesia. A gastroscope (GIF-HQ190; Olympus, Hamburg, Germany), a 1.5-mm ESD knife (Dualknife, Olympus), and a lifting gel (Eleview, Medtronic, Minneapolis, Minn, USA) were used. First, subepithelial injection and incision were performed distally, followed by proximal injection and incision. The dissection from the muscularis propria was difficult because of fibrosis, and we used a 9.5-mm traction magnet system (ProdiGi Traction Magnet, Medtronic) to expose the submucosa. This system is composed of 2 identical units. Each has a 9-mm routable clip and a neodymium magnet connected by a suture (Fig. 1). After passing the endoscope’s 2.8-mm working channel, the traction magnet was deployed onto the partially dissected tissue flap (Fig. 2). Afterward, the second unit was passed through the endoscope. The magnet of the second unit adhered to the first magnet, and the grasper of the second unit was attached to the targeted wall (Fig. 3). Butylscopolamine was administered to reduce GI motility, and air suction reduced the duodenal lumen, facilitating device deployment.

**Figure 1:**
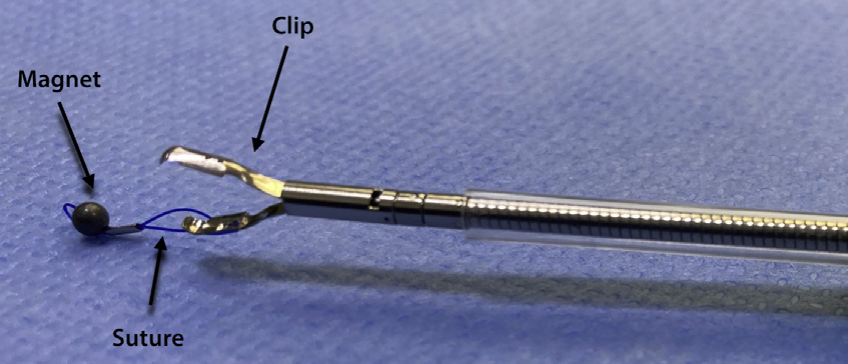
The traction system is composed of a 9-mm routable clip and a neodymium magnet connected by a suture.

**Figure 2:**
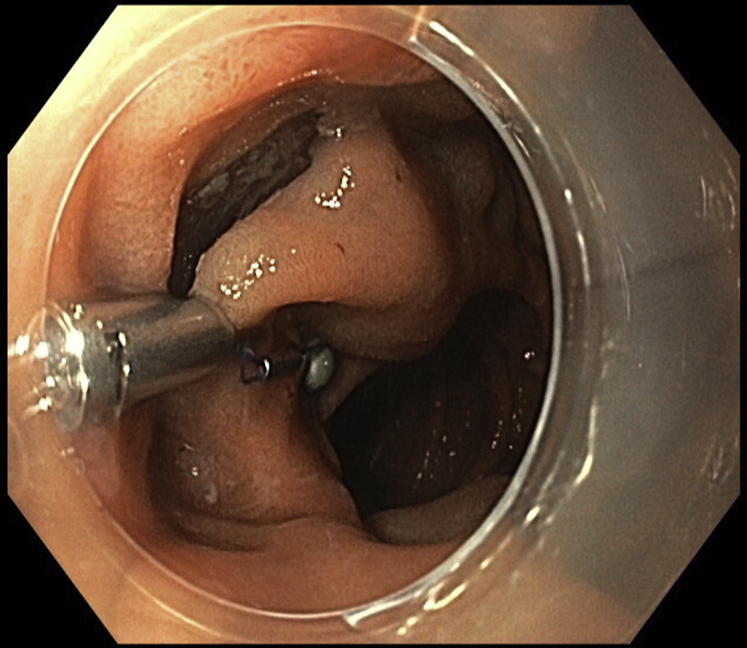
Deployment of the system in the proximal mucosal flap.

**Figure 3:**
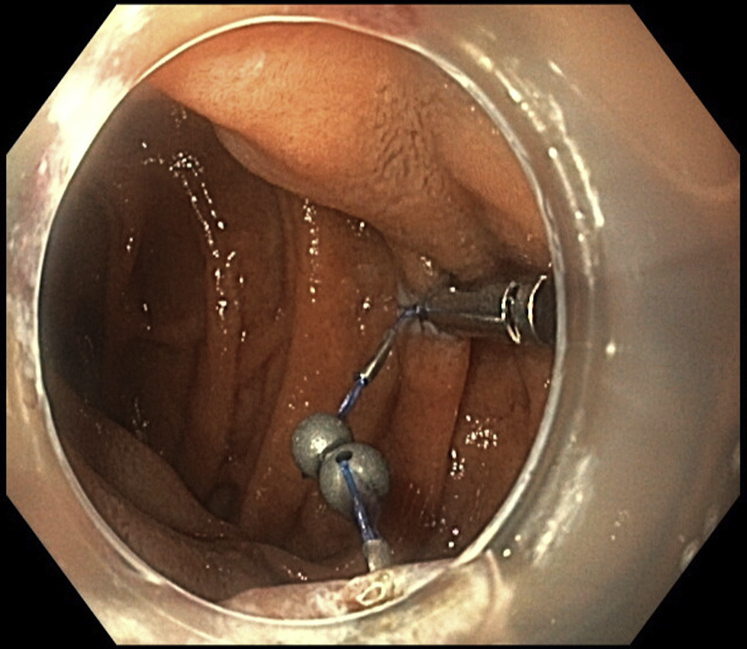
Placement of the additional system. The magnet of the second unit adhered to the first magnet, and the grasper of the second unit was attached to the targeted wall in the duodenum.

After partial dissection, there was the need for more traction, and a third magnet was used (Fig. 4). In this way, it was possible to obtain continuous tension during the ESD, achieving full resection with free margins. We used an endoscopic scissor (Loop cutter, Olympus) to cut the sutures in each unit. The specimen, 1 grasper, and 3 magnets were retrieved with a net (Fig. 5). There were no adverse events during the procedure, and the mucosal defect was closed using clips. The ESD took 69 minutes, and the endoscopy took 125 minutes.

**Figure 4:**
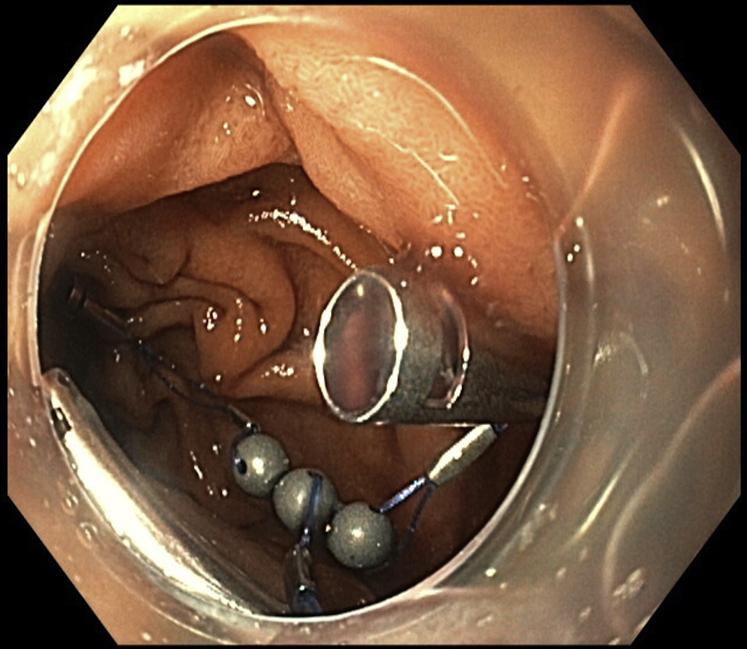
For further traction, a second system was used, with the clip deployed more distally in the duodenum, enabling traction.

**Figure 5:**
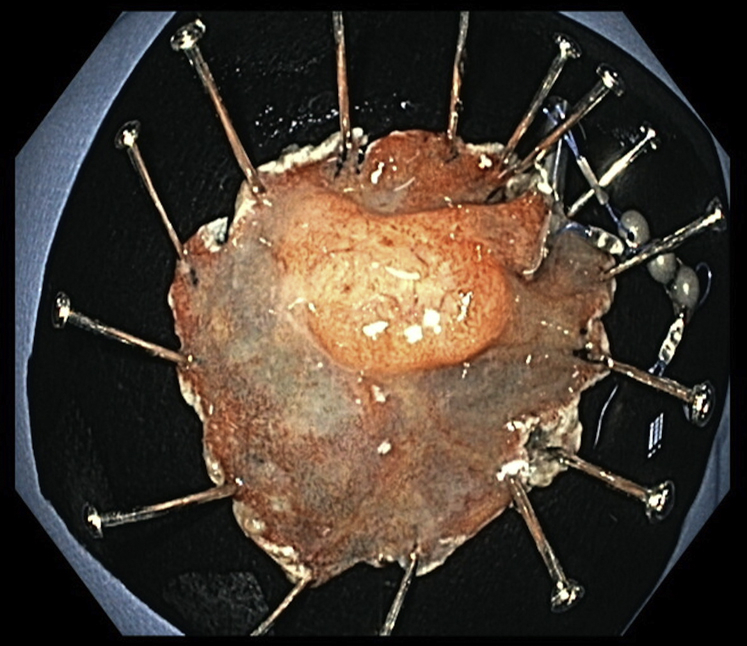
Full resection was obtained, and the specimen, 1 clip, and 3 magnets were retrieved using a net.

The pathology analysis showed a 28- × 24-mm fragment with a fully resected 12- × 6-mm neuroendocrine tumor, highly positive for chromogranin A, synaptophysin, and CDX2 and negative for somatostatin and serotonin. There were small segments without deep free margins owing to fulguration. The proliferative index was low (<1%), and a diagnosis of a well-differentiated neuroendocrine tumor (grade 1) was made (Fig. 6). The patient was discharged with no postoperative adverse events on day 2 and was asymptomatic at the 1-month follow-up (Video 1, available online at www.VideoGIE.org). Because there were no free deep margins in the resected specimen, a tight follow-up with EUS and endoscopy was proposed.

**Figure 6:**
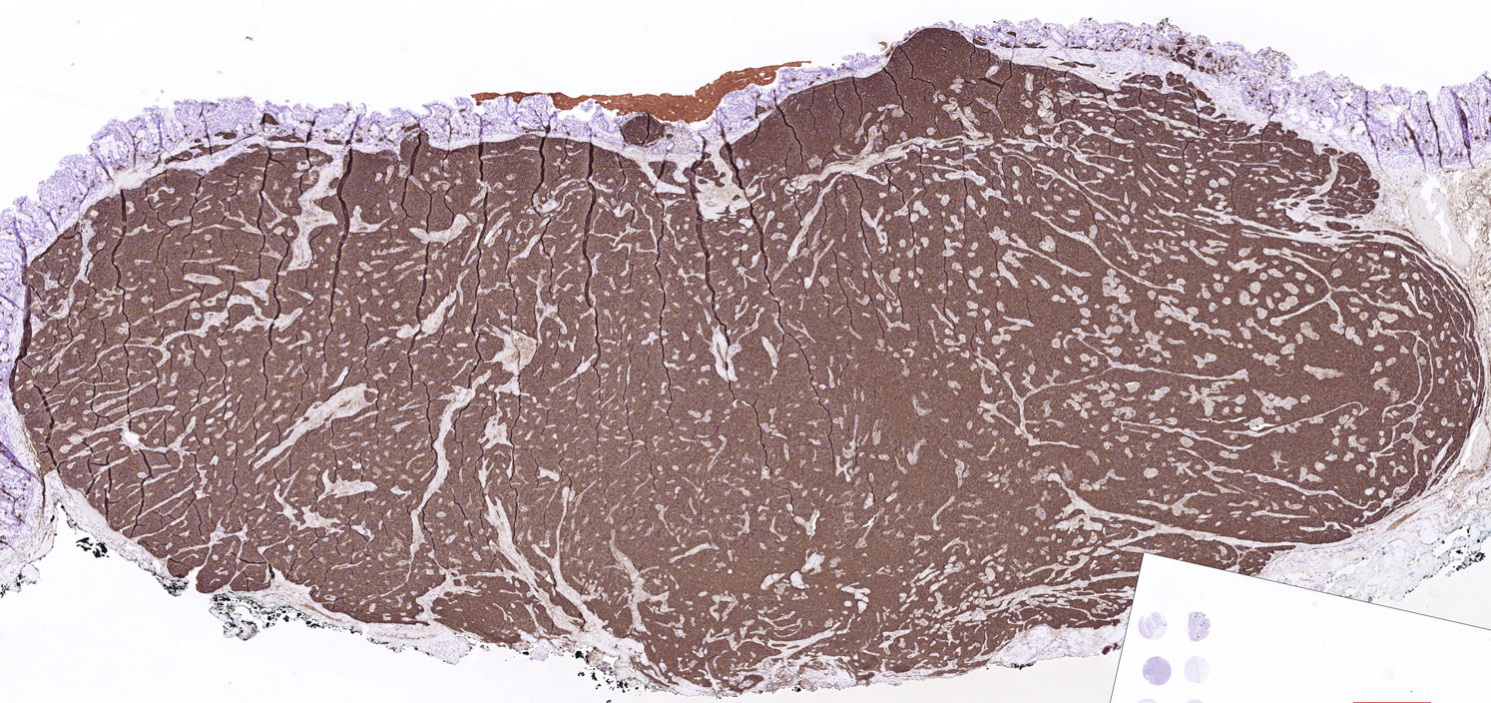
The pathologic investigation demonstrates the presence of a 28- × 24-mm fragment with a fully resected 12- × 6-mm neuroendocrine tumor, highly positive for chromogranin A.

Proper access to the submucosa layer is pivotal in ESD because it enables a faster and safer dissection. This is of paramount importance in difficult ESDs, namely lesions with fibrosis, subepithelial lesions, and in organs with a small lumen and thinner wall, such as the duodenum.^1^ Several devices and techniques have been developed to increase access to the submucosal layer. However, most have their limitations. Many enable traction in only 1 direction, and some are complex with the need for several steps, devices, and operators.^2^, ^3^, ^4^

We describe the use of a new traction device for the resection of a neuroendocrine tumor in the duodenum. This device may be deployed through a 2.8-mm working channel, and it enables internal traction (ie, there is no need for external magnets, strings, or snares). Continuous traction can be obtained by grasping more magnets to the dissecting tissue or adding more magnets to the previous ones. There is no need for extra personnel to handle the device after its deployment. In the duodenum, with its narrow lumen and thin wall, the deployment and use of larger devices might be hampered.^5^ This new magnet traction system, with its small components and flexibility, might be particularly useful in duodenal ESD.

Ingestion of magnets has been associated with adverse events such as volvulus and bowel perforation. Caution is recommended with this device, and it is necessary to remove the magnets at the end of the procedure. Removal can be done by using several devices, such as net, snare, cutter, or forceps.

In conclusion, this traction system is easy to deploy, user-friendly, and useful for difficult ESDs. Further comparative studies with other techniques or devices are warranted.

## Disclosure

*All authors disclosed no financial relationships.*

## Figures and Tables

**Video still:**
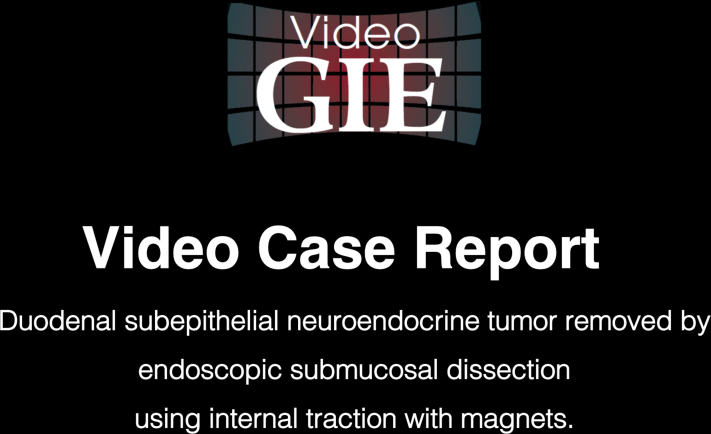


## Supplementary data

Video 1 Endoscopic submucosal dissection of a duodenal subepithelial neuroendocrine tumor using internal traction with magnets.
